# Anti-Inflammatory Potential of *Levilactobacillus brevis* LBH1070 and Its Synbiotic in a Murine Model of Experimental Arthritis

**DOI:** 10.3390/microorganisms14071473

**Published:** 2026-07-04

**Authors:** Morayma Ramírez-Damián, Cynthia Garfias-Noguez, Claudia Albany Reséndiz-Mora, María de Jesús Perea-Flores, Flor Nohemí Rivera-Orduña, Jorge Luis Gutiérrez-Ávila, Luis G. Bermúdez-Humarán, Gabriel Alfonso Gutiérrez-Rebolledo, María Elena Sánchez-Pardo

**Affiliations:** 1 Unidad Profesional Adolfo López Mateos, Escuela Nacional de Ciencias Biológicas, Instituto Politécnico Nacional, Zacatenco, Av. Wilfrido Massieu 399, Col. Nueva Industrial Vallejo, Alcaldía Gustavo A. Madero, Ciudad de Mexico C.P. 07738, Mexico; mramirezd1601@alumno.ipn.mx (M.R.-D.); cgarfez@gmail.com (C.G.-N.); cresendizm@ipn.mx (C.A.R.-M.); flor_1413@hotmail.com (F.N.R.-O.); jgutierreza2000@alumno.ipn.mx (J.L.G.-Á.); 2 Unidad Profesional Adolfo López Mateos, Centro de Nanociencias y Micro y Nanotecnologías, Instituto Politécnico Nacional, Zacatenco, Av. Luis Enrique Erro s/n, Col. Nueva Industrial Vallejo, Alcaldía Gustavo A. Madero, Ciudad de Mexico C.P. 07738, Mexico; mpereaf@ipn.mx; 3 Domaine de Vilvert, Micalis Institute, INRAE, AgroParisTech, Université Paris-Saclay, 78350 Jouy-en-Josas, France; luis.bermudez@inrae.fr

**Keywords:** arthritis, probiotic, synbiotic

## Abstract

Arthritis is a chronic inflammatory disease characterized by progressive joint damage, in which oxidative stress and exacerbated immune responses play a critical role. Synbiotics, defined as a combination of probiotics and prebiotics, may enhance these beneficial effects; however, their efficacy depends on maintaining microbial viability throughout gastrointestinal transit. In this study, we evaluated the anti-inflammatory and antioxidant effects of *Levilactobacillus brevis* LBH1070. *Lacticaseibacillus paracasei* ATCC 334 and phenylbutazone (PBZ) were used as probiotic and allopathic drug controls, respectively. Both probiotic strains significantly reduced paw edema by 46%, comparable to PBZ (45%), and decreased lipid oxidation (33–36%) and protein oxidation (40–45%), thereby preserving the integrity of the popliteal lymph node, the lymph node closest to the edema site. Furthermore, *L. paracasei* ATCC 334 and *L. brevis* LBH1070 reduced total T helper CD4+ lymphocyte infiltration in the popliteal lymph node by 44–54% and decreased IL-1β-producing T helper lymphocytes by 77–78%, surpassing the effect of PBZ (49%). TNF-α-producing T lymphocytes were also reduced by 60–62%, compared to PBZ (53%). These findings highlight the potential of *L. brevis* LBH1070, its synbiotic formulation, and *L. paracasei* ATCC 334 as complementary treatments to modulate immune responses and oxidative stress during arthritis.

## 1. Introduction

Arthritis (Ar) is a chronic autoimmune disease that primarily affects synovial joints and is characterized by persistent inflammation, pain, and progressive destruction of cartilage and bone, which can lead to severe functional disability [1,2]. Its etiopathogenesis involves a combination of genetic factors, such as the presence of the “shared epitope” on HLA-DR molecules, and environmental factors that promote inappropriate immune activation. This imbalance is reflected in excessive secretion of proinflammatory cytokines, including IL-1, IL-6, and TNF-α, along with reduced levels of anti-inflammatory cytokines such as IL-10 [3,4]. Major risk factors include smoking, infections, and, more recently, intestinal dysbiosis [5]. In addition, arthritis is associated with an oxidative stress process linked to chronic inflammation, which plays a key role in the development and pathophysiology of this chronic degenerative autoimmune disease [6,7].

The gut microbiota actively participates in the pathogenesis of arthritis through several mechanisms. One of them is the post-translational modification of host proteins, mediated by an increased abundance of oral bacteria in the gastrointestinal tract, such as *Aggregatibacter actinomycetemcomitans* and *Porphyromonas gingivalis*. These bacteria produce leukotoxin-A and the enzyme peptidyl arginine deiminase (PAD), leading to protein citrullination; the accumulation of citrullinated proteins triggers a dysregulated immune response against these autoantigens throughout the host body [8]. Another mechanism is molecular mimicry: some bacteria, particularly those belonging to the genus Prevotella, possess antigens structurally like the arthritis-associated citrullinated autoantigen N-acetylglucosamine-6-sulfatase (GNS), thereby activating and exacerbating T- and B-lymphocyte responses [4]. Additionally, experimental arthritis models have shown that bacteria such as *P. copri* and segmented filamentous bacteria (SFB) elicit inflammatory responses and promote Th17 and Th1 cell activation [3]. Finally, increased intestinal permeability associated with pathogenic bacteria fosters inflammatory cell recruitment and generates colitis development [3,4,8].

Recent studies have shown that patients with arthritis exhibit an altered microbial composition, characterized by increased intestinal abundance of Bacteroides, Escherichia, Shigella, and Prevotella species, particularly *P. copri*, which is associated with heightened inflammation and disease severity. In contrast, genera such as *Lactobacillus spp*. and *Bifidobacterium* are reduced in abundance [5,9]. Moreover, compared with healthy individuals, arthritis patients exhibit elevated levels of Verrucomicrobia, Lactobacillus, Streptococcus, Akkermansia, and Proteobacteria, along with decreased levels of Bacteroidetes, Bacteroides, and Faecalibacterium. Cytokine profiles are also altered, with elevated concentrations of TNF-α, IL-6, IL-10, IL-4, and IL-2 [10].

Given these findings, modulation of the gut microbiota using probiotics has gained significant interest. Probiotics—defined as live microorganisms that, when administered in adequate amounts, confer health benefits [11]—have demonstrated immunomodulatory effects in arthritis. Clinical trials involving strains such as *Lactobacillus rhamnosus*, *L. casei*, *Bacillus coagulans*, *L. reuteri*, *L. acidophilus* and *Bifidobacterium bifidum* have shown improvements in gastrointestinal symptoms and immune function [1,12,13,14,15].

Furthermore, the growth and efficacy of probiotics can be enhanced when combined with prebiotics, non-digestible substrates such as inulin and galactooligosaccharides (GOS), which selectively stimulate beneficial bacteria and promote short-chain fatty acid (SCFA) production [16,17]. Prebiotics support probiotic survival, thereby improving gut microbiota composition, intestinal metabolic activity, and preventing pathogenic bacterial growth. Consequently, the simultaneous consumption of probiotics and prebiotics may yield greater health benefits, including reduced levels of undesired metabolites, inhibition of carcinogenic substances and nitrosamines, and increased production of SCFAs, carbon disulfides, methyl acetates, and ketones [16,17,18]. This complementary relationship forms synbiotics, defined by the International Scientific Association for Probiotics and Prebiotics (ISAPP) as mixtures of live microorganisms and one or more substrates selectively utilized by host microorganisms that confer health benefits [19].

A key challenge in the application of probiotics and synbiotics is the limited viability of probiotic bacteria under gastric, biliary, and industrial processing conditions [20,21]. In this context, microencapsulation represents an effective strategy to enhance probiotic stability by protecting them from environmental stress (such as exposure to gastric juice), promoting controlled intestinal release, and allowing the production of synbiotic formulations. Among the available techniques, spray drying has proven effective at the industrial level, particularly when using coating materials such as maltodextrin, gum Arabic, and inulin, which additionally provide prebiotic properties [22,23,24,25].

Currently, standard arthritis treatment is based on a pyramid and polypharmacy scheme, starting in Ar early stages with Non-Steroidal Anti-Inflammatory Drugs (NSAIDs), which are non-selective inhibitors of both cyclooxygenases isoforms (1 cytoprotective and 2 inflammatory); when the inflammation worsens, an NSAID is combined with a disease-modifying antirheumatic drugs (DMARDs), such as methotrexate [26]. On the other hand, phenylbutazone, a veterinary NSAID used in preclinical models for anti-arthritic evaluation, not only inhibits cyclooxygenases but also has an immunosuppressive effect like DMARDs [27]. However, 20–30% of patients discontinue treatment during the first year due to adverse effects, including nausea, vomiting, diarrhea, gastroduodenal ulcers, gastric bleeding, hepatotoxicity, nephrotoxicity, pulmonary complications, immunosuppression, opportunistic infections, and hair loss [1] Although combining TNF-α inhibitors with methotrexate may reduce discontinuation rates, TNF-α inhibitors are also associated with an increased risk of pneumonia and bacterial, viral, or fungal infections, particularly in the respiratory, urinary, gastrointestinal tracts, as well as the skin [2,9]. Given the potential adverse effects of allopathic anti-inflammatory drugs and the role of environmental factors in genetically predisposed individuals, dietary interventions and probiotic supplementation should be considered as alternative or complementary strategies for controlling the development of arthritis [5,28]. This has driven the exploration of safer complementary approaches, including modulation of the microbiota through probiotics and synbiotics.

Among probiotics of interest, *Lactobacillus brevis* has been studied for its biotechnological relevance and immunomodulatory potential. In particular, the strain *L. brevis* LBH1070, isolated from pulque in Milpa Alta, Mexico, has been shown to adhere to intestinal epithelial cells and reduce proinflammatory cytokine production *in vitro* [29].

Overall, the available evidence suggests that probiotics and synbiotics can help restore gut microbiota and reduce inflammation in arthritis. In this study, we evaluated the anti-inflammatory, antioxidant, and immunomodulatory effects of *L. brevis* LBH1070 and its microencapsulated synbiotic formulation in a murine model of experimental arthritis, considering the key role of oxidative stress in disease pathophysiology [6,7]. This work is relevant both for its technological and biotechnological innovation and for its potential impact on the prevention and treatment of chronic diseases using autochthonous microorganisms with functional properties.

## 2. Materials and Methods

### 2.1. Biological Material

A potentially probiotic strain previously isolated by Torres-Maravilla et al. [29] from pulque made in Milpa Alta, Mexico City, Mexico, was used in this study. As a probiotic control, the commercial strain *Lacticaseibacillus paracasei* ATCC 334 was provided by the French National Research Institute for Agriculture, Food and Environment (Centre de recherche INRAE Île-de-France, 4 rue Jean Jaurès, 78352 Jouy-en-Josas Cedex, France). The synbiotic formulation was produced by spray drying using maltodextrin (5%, MD) and gum Arabic (10%, GA) as wall materials, and inulin (5%, I) as a prebiotic, as described by Ramírez-Damián et al. [30]. The proportions of MD, GA, and I used in the formulation were determined from unpublished preliminary work reported in European Patent Application No. EP24306864.0.

### 2.2. In Vitro pH Tolerance Test

According to Castillo Arroyo et al. [31], *Lactobacillus* strains were inoculated at 1% (*v*/*v*) for free strains or 1% (*w*/*v*) for microencapsulated strains into MRS broth at pH values ranging from 1 to 8. The pH was adjusted using 1N HCl and 1N NaOH. Determinations were performed in triplicate. After 24 h of incubation at 37 °C, growth was measured by determining optical density at 600 nm. The test was carried out in triplicate (*n* = 3) and the result was reported as the % of tolerance to a certain pH, calculated using the following Equation (1):

% pH tolerance = [OD_MRS_ at different pH/OD_MRS_ at basal pH] × 100 (1)

### 2.3. Experimental Animals’ Maintenance Conditions

A total of 60 male CD-1 mice (25 ± 5 g) were acclimated for 7 days prior to the start of the experiment. Animals were housed under a 12 h light/dark cycle at 24 ± 2 °C, with 70% relative humidity, and had free access to standard rodent diet (Rodent Diet 5001, LabDiet, Hubbard, OR, USA) and water. All procedures followed the guidelines of the International Committee for the Care and Use of Laboratory Animals (CICUAL) and the Mexican Official Standard NOM-062- ZOO-1999 entitled: “Technical Specifications for the Reproduction, Care and Use of Laboratory Animals” and modified in 2001 [32]. The study was carried out on male CD1 mice, because previous work has shown that under similar conditions of CFA-induced experimental arthritis in different strains, males were able to maintain a sustained inflammatory response for up to 32 days during monoarthritis, unlike females, who were resistant to keeping the plantar edema beyond 10 days [33].

### 2.4. Experimental Arthritis

The murine model of experimental arthritis was induced using Complete Freund’s Adjuvant (CFA), as described by Gutiérrez-Rebolledo et al. [34].

**Figure 1:**
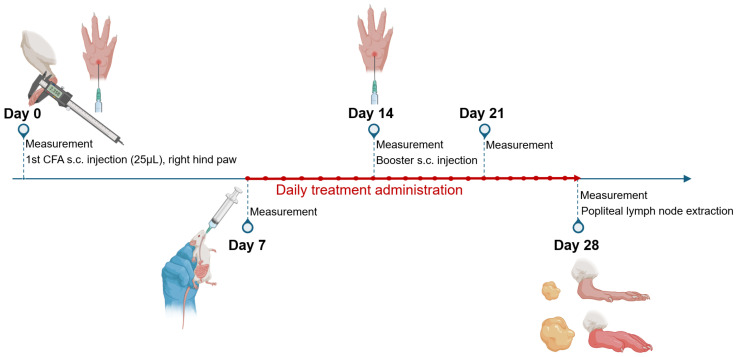
**Experimental design of CFA-induced arthritis model.** Mice received subcutaneous (s.c) CFA injections on days 0 and 14; treatments were administered intragastrically (i.g.) from day 7 to day 27 (red line); paw edema was measured on days 7, 14, 21, and 28; and animals were euthanized on day 28 for lymph node collection. CFA: Complete Freund’s Adjuvant; s.c.: subcutaneous, i.g.: intragastric.

Mice were randomly assigned to six groups (*n* = 10): (1) healthy control; (2) arthritic control (untreated); (3) arthritic + phenylbutazone (100 mg/kg in tween80:water, 1:9); (4) arthritic + reference probiotic (*L. paracasei* ATCC 334, 1 × 10^7^ CFU/mL); (5) arthritic + *L. brevis* LBH1070 (free cells, 1 × 10^7^ CFU/mL); (6) arthritic + synbiotic (*L. brevis* LBH1070 microencapsulated in MD, GA, and I, 1 × 10^7^ CFU/mL). Groups 2–6 received subcutaneous CFA injections (25 μL) into the right hind paw on days 0 and 14. Treatments (groups 3–6) were given intragastrically from day 7 to 27 (10 mL/kg), while controls (groups 1 and 2) received water. The experimental design appears in Figure 1.

Subplantar edema development was measured on days 7, 14, 21, and 28 (E_t_) using a digital micrometer (Mitutoyo, model 293-831, Kawasaki, Japan), with measurements on day 0 (E_0_) used as baseline. Edema inhibition (%) was calculated relative to the untreated arthritic control group (group 2).

% edema inhibition = [(E_t_ − E_0_) CFA un-treated − (E_t_ − E_0_) CFA treated/(E_t_ − E_0_) CFA un-treated] × 100 (2)

On day 28, body weight was recorded, and mice were euthanized by cervical dislocation. Subsequently, the popliteal lymph node closest to the site of subplantar edema was excised: one lymph node was used for histological analysis (*n* = 3), and six were used for oxidative stress analysis and immune cell quantification (*n* = 7).

#### 2.4.1. Histology

Popliteal lymph nodes were collected for histological evaluation (*n* = 3 per group, 5 slices per sample), following the methodology of Gutiérrez-Rebolledo et al. [34]. Samples were embedded in Tissue-Tek (Sakura Finetek, Torrance, CA, USA), frozen, and stored at −70 °C. Tissues were then sectioned at 6 μm using a rotary microtome with a freezing system (Leica CM1900, Wetzlar, Germany). Sections were stained with hematoxylin and eosin, fixed in 2% formaldehyde for 1 h at room temperature, and washed with PBS (pH 7.4). Histological evaluation was performed using a light microscope (Leica White MP32, Wetzlar, Germany) to identify tissue alterations by comparison with the healthy and untreated arthritic control groups.

The descriptive macroscopic evaluation of the stained sections from each group (*n* = 3) was complemented with a subjective Histological Damage (HD) evaluation with the assignment of a numerical value based on the following criteria: oxidative and inflammatory damage was assessed on a scale from 0 to 3: (0) no infiltrated cells/lipid vacuoles with continuity of the lymph node tissue, (1) few infiltrated cells and small lipid vacuoles with tissue continuity, (2) moderate infiltrated cells and medium lipid vacuoles with discontinuous tissue, and (3) increased infiltrated cells and large lipid vacuoles that have disrupted the tissue [35].

#### 2.4.2. Oxidative Stress

Popliteal lymph node tissues (*n* = 7 per group) were weighed and homogenized in an ice bath using 1 mL of cold PBS (pH 7.4). From the resulting homogenate, 500 µL were used for lipid peroxidation (LPx) analysis, 300 µL for protein oxidation (POX) determination, and the remaining 200 µL were centrifuged at 12,500 rpm for 45 min at 4 °C. The supernatant was collected to assess antioxidant enzyme activities, including superoxide dismutase (SOD) and glutathione peroxidase (GPx).

##### Lipid Peroxidation Rate

An aliquot of 500 µL of lymph node homogenate was mixed with 2 mL of TBARS reagent (16% trichloroacetic acid, 0.5% thiobarbituric acid, 0.3 N HCl in deionized water), vortexed for 5 min, and incubated at 80 °C for 15 min. Samples were then cooled on ice for 15 min and centrifuged at 4000 rpm for 10 min at 4 °C. The absorbance of the supernatant was measured at 535 nm. TBARS reagent processed under the same conditions but without homogenate was used as a blank [7]. MDA levels were expressed as micromoles per gram of lymph node tissue (MDA µmol/g tissue) and calculated using a molar extinction coefficient of 1.56 × 10^5^ M^−1^ cm^−1^.

##### Protein Oxidation

An aliquot of 300 µL of lymph node homogenate was mixed with 300 µL of 20% trichloroacetic acid (TCA), vortexed, and incubated on ice for 15 min. Samples were centrifuged at 11,495 rpm (3000× *g*) for 5 min at 4 °C. The resulting precipitate was resuspended in 150 µL of 2,4-dinitrophenylhydrazine (2,4-DNPH, prepared in 2 M HCl), vortexed for 5 min, and incubated at 37 °C for 1 h in the dark. After incubation, samples were centrifuged at 10,000× *g* for 10 min at 4 °C, the supernatant was discarded, and the pellet was washed three times with 1 mL of ethyl acetate–ethanol mixture (1:1, *v*/*v*). The final precipitate was dissolved in 1 mL of 6 M guanidine, prepared in 0.2 mM potassium phosphate buffer (pH 2.3), vortexed for 5 min, and incubated for 40 min at 37 °C in the dark. Samples were then centrifuged at 3000× *g* for 5 min at 4 °C, and the absorbance of the supernatant was measured at 360 nm. The reagent blank consisted of 1 mL of guanidine solution without a sample. Protein oxidation was expressed as micromoles of reactive carbonyls per gram of lymph node tissue (CO● µmol/g tissue), calculated using a molar extinction coefficient of 21,000 M^−1^ cm^−1^.

##### Superoxide Dismutase Activity

SOD activity was assessed using the RANSOD kit (No. CAT SD125, RANDOX, UK), according to the manufacturer’s instructions. Briefly, 50 µL of lymph node homogenate supernatant was mixed with 1.7 mL substrate (R1) and 0.25 mL xanthine oxidase (R2), vortexed, and incubated at 37 °C for 30 s. Absorbance was measured at 505 nm immediately (A_1_) and after 3 min (A_2_). SOD activity was calculated using a standard curve (following kit instructions) and expressed as UI SOD/g tissue (popliteal lymph node).

##### Glutathione Peroxidase Activity

GPx activity was determined using the RANSEL kit (No. CAT RS504, RANDOX, UK) following the manufacturer’s protocol. A total of 20 µL of lymph node homogenate supernatant was mixed with 1 mL reagent (R1) and 40 µL cumene hydroperoxide (R2), vortexed, and incubated at 37 °C for 1 min. Absorbance was measured before (A_1_) and after 1 min incubation at 37 °C (A_2_). GPx activity was expressed as UI GPx/g tissue (popliteal lymph node).

#### 2.4.3. Immunomodulatory Activity

**Figure 2:**
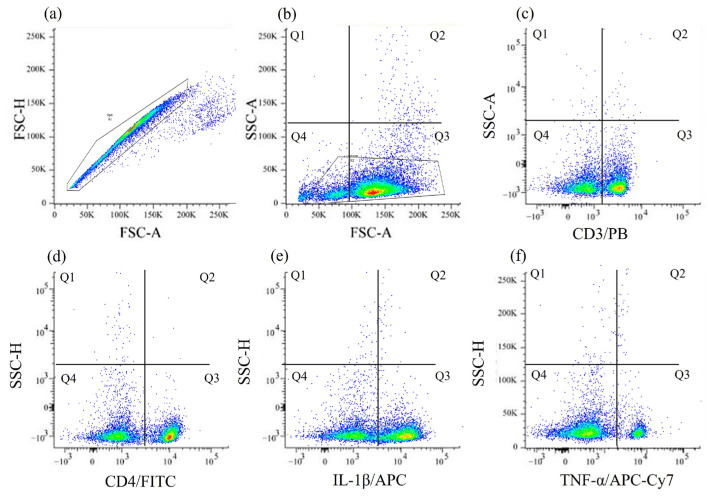
**Flow cytometer strategy.** (**a**) Selection of ‘singlet’ events. (**b**) Selection of events by size vs. intracellular granularity/complexity. (**c**) Selection of events within the chosen size and granularity to show the presence of T lymphocytes (CD3/PB). (**d**) Selection of events within CD3 events to show which T lymphocytes were “helpers” (CD4/FITC). (**e**,**f**) Selection of events within CD4 events to show which produce IL-1β (APC), and TNF-α (APC-Cy7). Q1, Q2, Q3, and Q4 from (**b**–**e**) indicate the chosen quartiles to separate, group, and analyze each of the present subpopulations, while the color scale lets you check the abundance of a subpopulation based on its immunofluorescence (red = very abundant, blue = not very abundant).

Popliteal lymph nodes were mechanically dissociated in a ground glass mortar under freezing conditions using 1 mL of cold PBS/FACS buffer (pH 7.4). Cells were stained for extracellular (CD3^+^, CD4^+^) and intracellular (TNF-α, IL-1β, IL-10) markers using anti-mouse CD3^+^/PB (identify T lymphocytes), and CD4^+^/FITC (to identify only T lymphocytes “helpers” secretors of IL’s), in mix, and then anti-mouse TNF-α/APC-Cy7, anti-mouse IL-1β/APC, and anti-mouse IL-10/PE antibodies in mix [34]. Compensation controls were prepared and analyzed for each antibody, along with unstained cells, to adjust and calibrate the cytometer before sample analysis. Samples were stained using the antibody mixture for both extracellular and intracellular markers. Flow cytometry analysis was performed using a CytoFLEX cytometer (Beckman Coulter, B5V5R3 CA, USA) and data were analyzed with FlowJo software version 10.0.6 (Tree Star, Ashland, OR, USA), according to what is shown in the flow cytometer strategy (Figure 2). All antibodies were purchased from eBioscience (San Diego, CA, USA). Absolute values of infiltrating T lymphocyte subpopulations in lymph node tissue were calculated based on the relative percentages obtained by flow cytometry and total cell counts determined using a Neubauer chamber. Results were expressed as the total number of cells × 10^6^ per lymph node.

### 2.5. Statistical Analysis

All results are expressed as mean ± standard error of the mean (SEM). Graphs and statistical analyses were performed using GraphPad Prism version 8 (GraphPad Software, Boston, MA, USA). For variables that met parametric assumptions, a one-way analysis of variance (ANOVA) with the Student–Newman–Keuls (SNK) post hoc test was used. For pH tolerance test (%) and relative popliteal lymph node weight (%), which did not meet parametric assumptions, the Kruskal–Wallis test followed by Dunn’s post hoc test was applied. Differences were considered statistically significant at *p* < 0.05.

## 3. Results and Discussion

### 3.1. Tolerance to Acidic or Alkaline Conditions In Vitro

Currently, a large number of both preclinical and clinical studies have shown that the gut microbiota exerts beneficial molecular and cellular mechanisms by regulating preexisting pathophysiological processes or delaying/preventing their manifestation, not only locally but systemically, which is why it is considered a “second brain,” exerting therapeutic effects like anti-inflammatory action during chronic colitis models in rodents, and in patients with autoimmune diseases like Crohn’s [36]. The free *Levilactobacillus brevis* LBH107 and microencapsulated strains evaluated showed good resistance across a range of pH values, including low pH. Although survival rates decreased under acidic conditions, they remained above 40% (Table 1).

It has been reported that Lactobacillus strains can survive 41% to 89.93% after 180 min at pH 2 [29]. Similarly, *L. rhamnosus*, *L. casei*, and *L. paracasei* survived for 2 h at pH 1.5 and 2.5 [37]. In another study, *Lactobacillus acidophilus* NCIM 2660, *Lactobacillus bulgaricus* NCIM 2056, and *Lactobacillus fermentum* NCIM 2165 showed survival rates of 24%, 38%, and 34%, respectively, after 2 h at pH 2 [38].

Other authors have also reported greater resistance of microencapsulated strains at low pH; for example, *Lactobacillus acidophilus* La-5 showed a survival rate of 23.55% in free form, compared to 77% when microencapsulated, after exposure to simulated gastric conditions at pH 2 [39]. Likewise, after 120 min under simulated gastric conditions, the cell count of free *L. plantarum* BM-1 decreased by approximately 1.4 log CFU/mL (~83% of the initial count), whereas the microencapsulated strain showed no significant reduction [40]. Meanwhile, survival rates of 15% and 62% after 3 h at pH 1, 19% and 68% at Lactobacillus pH 2 were observed for *Lactobacillus acidophilus* NCDC 016 in its free and microencapsulated forms, respectively [41].

Therefore, the survival of strain LBH1070 under simulated low-pH conditions, even in its free form, suggests good tolerance to gastrointestinal pH variations, an essential characteristic for selecting strains with probiotic potential, that could regulate inflammatory processes.

### 3.2. Anti-Edema Effect and Relative Weight of Lymph Nodes

The search for new anti-arthritic treatments has increased along with the prevalence and incidence of this complex autoimmune disease and its pathophysiology, which makes it challenging to fully replicate it preclinically; however, there are currently murine models that are validated and well-characterized in their pathophysiology, such as those induced by CFA in rodent’s paw. Although it has the limitation of being very localized, the observed chronic inflammatory condition exacerbating not only synovial or sub plantar edema, but also provoking systemic erythema, hyperalgesia, and even increased concentrations of proinflammatory cytokines not only in tissue but also in serum after paw s.c. injection [42]. Another limitation of this model is that in female mice there is greater variability in response due to physiological regulations dependent on sex hormones [33], so the aim of this study is to evaluate the redox and immune environment in the lymphatic tissue in arthritic male CD1 male.

**Figure 3:**
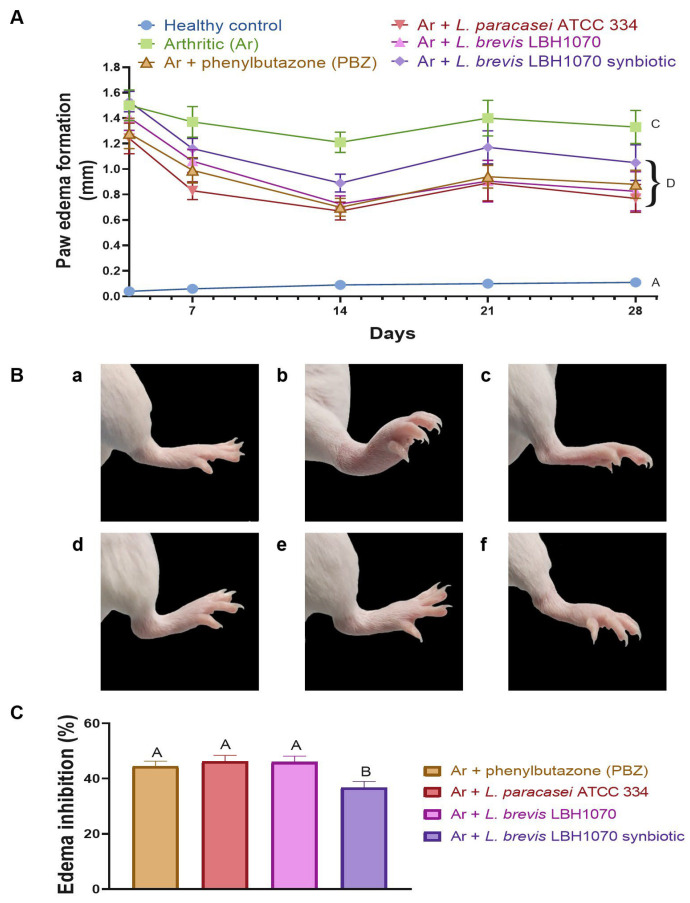
**Effect of treatments on paw edema inhibition on CFA-induced arthritic mice.** (**A**) Edema size. (**B**) Representative paw photographs: (**a**) healthy control, (**b**) arthritic control (Ar), (**c**) phenylbutazone (PBZ) (**d**) *L. paracasei* ATCC 334, (**e**) *L. brevis* LBH1070, (**f**) *L. brevis* LBH1070 synbiotic. (**C**) Edema inhibition on day 28 (%). Data are presented as mean ± standard error of the mean (SEM). Different uppercase letters indicate significant differences among treatments (ANOVA, Student–Newman–Keuls, *p* ≤ 0.05; *n* = 7).

In this experimental pathophysiological context, animals from the arthritic control group (Ar, CFA-induced, untreated) showed a significant increase in paw edema (1.33 ± 0.06 mm), calculated as the means of measurements recorded throughout the entire experimental period, compared to the healthy control group (0.08 ± 0.02 mm; *p* < 0.05). All treatments significantly reduced this increase, maintaining an average paw edema of 1.00 ± 0.03 mm above healthy control values throughout the model, with no significant differences among treatments (Figure 3A).

Qualitatively, visible differences in paw inflammation were also observed on day 28 (Figure 3B). The arthritic control group exhibited marked edema, in contrast to the healthy control group, which showed normal paw growth. Treatment with phenylbutazone (PBZ) produced the most pronounced reduction, resembling the healthy control, while the groups treated with *L. paracasei* ATCC 334, *L. brevis* LBH1070, and the *L. brevis* LBH1070 synbiotic also exhibited noticeable improvements, although moderate inflammation remained.

Edema inhibition on day 28 was 45% in the PBZ-treated group, with no significant differences compared to *L. paracasei* ATCC 334 (46%) and *L. brevis* LBH1070 (46%) (*p* < 0.05). These findings suggest that free-form probiotics are as effective as the anti-inflammatory drug in reducing edema. In contrast, the *L. brevis* LBH1070 synbiotic showed significantly lower efficacy, with 15% less inhibition compared to its free form (Figure 3C).

**Figure 4:**
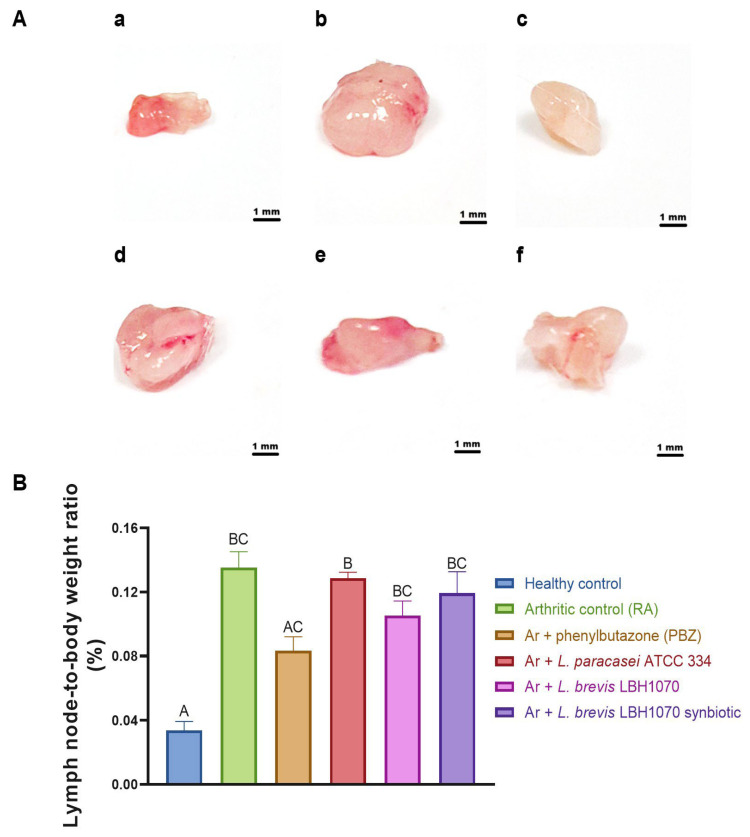
**Effect of treatments on popliteal lymph node size of CFA-induced arthritic mice on day 28.** (**A**) Representative photographs of excised popliteal lymph nodes: (**a**) healthy control, (**b**) arthritic control (Ar), (**c**) phenylbutazone (PBZ), (**d**) *L. paracasei* ATCC 334, (**e**) *L. brevis* LBH1070, (**f**) *L. brevis* LBH1070 synbiotic. (**B**) Relative lymph node weight to body weight. Data are presented as mean ± standard error of the mean (SEM). Different uppercase letters indicate significant differences among treatments (Kruskal–Wallis, Dunn, *p* ≤ 0.05; *n* = 7).

Similarly, the un-treated arthritic control group showed a marked increase in popliteal lymph node size, with relative weight reaching 0.14% of total mouse body weight on day 28 (38.4 g), compared to 0.03% in the healthy control group (*p* < 0.05) on same day (36 g). PBZ treatment significantly reduced this value to 0.08% (35.76 g) compared to arthritic control group, although it did not reach the values observed in the healthy control group. In contrast, *L. paracasei* ATCC 334, *L. brevis* LBH1070, and its synbiotic showed only modest reductions (0.11, 0.11, and 0.13%, respectively), with no significant differences compared to the arthritic control group (Figure 4A,B) at the end of the experiment (34, 33.84, and 34.35 g, respectively). Overall, these results indicate that the evaluated probiotics are effective in reducing edema to a similar extent as the anti-inflammatory drug; however, they do not significantly decrease the relative popliteal lymph node enlargement associated with chronic arthritis-related inflammation.

These findings indicate that *L. casei* ATCC 334, *L. brevis* LBH1070, and its synbiotic are capable of significantly reducing inflammation and swelling of the extremities in this experimental model of CFA-induced arthritis. These results are consistent with previous reports. For example, *Bacillus coagulans* with inulin significantly reduced paw inflammation and thickness in male Wistar rats with CFA-induced arthritis, demonstrating preventive and therapeutic effects comparable to those of the reference drug indomethacin [43]. Likewise, administration of *L. casei* ATCC 334 in Sprague-Dawley rats with CFA-induced arthritis significantly reduced joint inflammation and paw volume after 30 days of treatment [44]. In another study, *Bifidobacterium breve* NCIM 5671, *Bifidobacterium longum* NCIM 5672, and *Bifidobacterium bifidum* NCIM 5697 slowed the progression of inflammation in Wistar rats with CFA-induced arthritis, resulting in significantly less paw swelling compared to untreated animals [45]. Finally, male BALB/c mice with collagen antibody-induced arthritis (CAIA) treated with *Bacillus coagulans* BACO 17 showed a marked reduction in paw swelling compared to untreated mice [46].

### 3.3. Popliteal Lymph Node Histology

**Figure 5:**
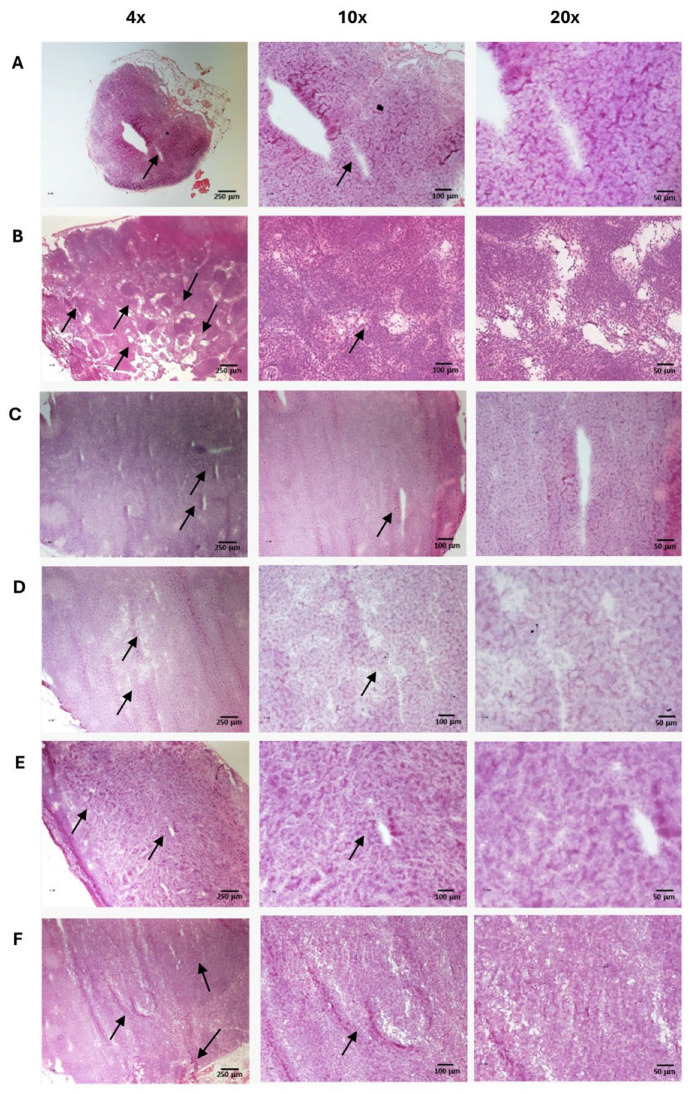
**Microphotographs of popliteal lymph nodes tissue from CFA-induced arthritic mice**. (**A**) Healthy control, (**B**) arthritic control, (**C**) phenylbutazone (PBZ), (**D**) *L. paracasei* ATCC 334, (**E**) *L. brevis* LBH1070, (**F**) *L. brevis* LBH1070 synbiotic. (*n* = 3 per group; 5 slices per sample). The staining of the sections corresponds to a normal result of the hematoxylin and eosin staining technique; the black arrows indicate the number of areas that represent lipid vacuoles of free fatty acids, showing tissue damage from lipid peroxidation, also being the areas that were maximized for a more detailed analysis.

Histological analysis of popliteal lymph node tissue from each experimental group revealed notable differences compared with healthy control (Figure 5). In the health control group (Figure 5A), popliteal lymph nodes appeared smaller and exhibited a uniform tissue architecture, reflecting preserved membrane integrity. Some vacuolated areas were observed, representing basal lipid peroxidation in tissues under physiological homeostasis. The HD score assigned to this group was just zero, since despite the vacuoles present, there was no noticeable leukocyte infiltration. In contrast, popliteal lymph nodes from the arthritic control group (Figure 5B) showed increased size and intense leukocyte infiltration. In addition, multiple vacuolated areas were observed throughout the tissue (highlighted in red), indicative of structural damage associated with lipid oxidation. This process involves the degradation of membrane phospholipids into free fatty acids—which accumulate in cytoplasmic vacuoles that do not interact with hematoxylin—and eosin dyes due to their neutral charge, suggesting deterioration of tissue integrity. Based on this macroscopic analysis, the HD score assigned to this group was 3 for even showing total disruption of the lymph node tissue.

In the PBZ-treated group (Figure 5C), although popliteal lymph node size remained comparable to that of the arthritic control group, a clear reduction in lipid inclusions was evident, suggesting a lower degree of tissue alteration and lipid peroxidation induced by the phagocytic respiratory burst. This effect can be attributed to the anti-inflammatory and immunosuppressive actions of PBZ, which includes inhibition of cyclooxygenase-1 (COX-1) and cyclooxygenase-2 (COX-2), resulting in reduced prostaglandin synthesis involved in vasodilation, pain, and immune cell recruitment to inflammatory sites. In addition, PBZ interferes with leukocyte maturation, thereby limiting macrophage activation and contributing to leukopenia and agranulocytosis during chronic inflammation [47]. Due to this mechanism of action, PBZ is assigned an HD score of 2 since the lymphatic tissue maintained its continuity to a greater extent despite the initiated lipid peroxidation process.

Comparable histological improvements were also observed in the groups treated with *L. paracasei* ATCC 334 (Figure 5D), *L. brevis* LBH1070 (Figure 5E), and the *L. brevis* LBH1070 synbiotic (Figure 5F), where tissue structure appeared better preserved with fewer vacuolated regions, despite maintaining a popliteal lymph node size similar to that of the untreated arthritic control group. Finally, based on their macroscopic evaluations, the probiotics showed the following HD values: arthritic mice treated with *L. paracasei* ATCC 334 scored 1, *L. brevis* LBH1070 scored 2, and *L. brevis* LBH1070 synbiotic scored 2.

The protective role of probiotics against inflammatory tissue damage in murine arthritis models has been previously documented. For instance, reduced inflammatory cell infiltration and decreased joint destruction were reported in DBA/1J mice with type II collagen-induced arthritis (CIA) treated with heat-killed *Lactobacillus reuteri* [48]. Similarly, histological sections of knee joints from Sprague-Dawley rats with adjuvant-induced arthritis (AIA) treated with *L. casei* ATCC 334 revealed reduced tissue damage [44]. Moreover, significant decreases in cartilage degradation were observed in the knee joint tissues of male BALB/c mice with collagen antibody-induced arthritis (CAIA) treated with *Bacillus coagulans* BACO-17 [46].

### 3.4. Oxidative Stress Biomarkers

**Figure 6:**
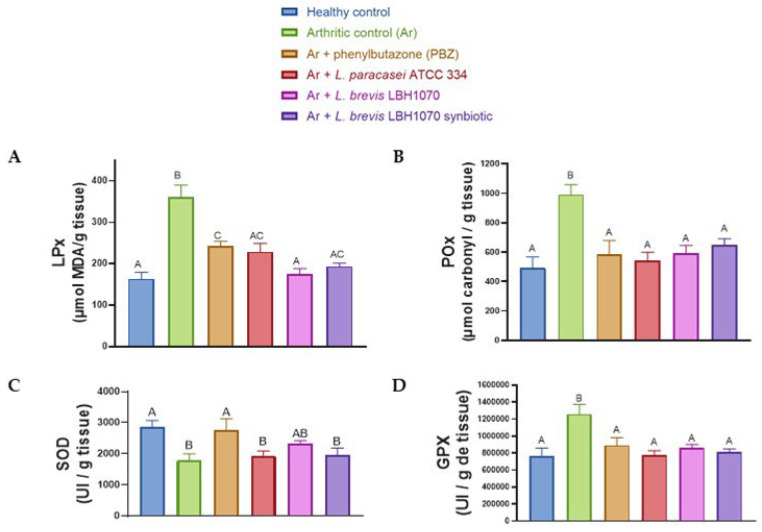
**Effects of treatments on oxidative stress in popliteal lymph nodes of CFA-induced arthritic mice**. (**A**) lipid peroxidation (MDA, µmol/g tissue), (**B**) protein oxidation (reactive carbonyls, µmol/g tissue), (**C**) superoxide dismutase activity (SOD, U/g tissue), (**D**) glutathione peroxidase activity (GPx, U/g tissue). Data are presented as mean ± standard error of the mean (SEM). Different uppercase letters indicate significant differences among treatments (ANOVA, Student-Newman-Keuls, *p* ≤ 0.05; *n* = 7).

Lipid and protein oxidation levels in popliteal lymph node tissue were significantly higher in the arthritic control group (362 µmol MDA/g tissue and 992 µmol CR/g tissue, respectively), representing approximately a two-fold increase compared with the basal levels observed in the healthy control group (163 µmol MDA/g tissue and 495 µmol CR/g tissue, respectively; *p* < 0.05; Figure 6A,B).

PBZ treatment significantly reduced arthritis-associated lipid and protein oxidation in the popliteal lymph node tissue, compared with the arthritic control group, by 32.6% (244 µmol MDA/g tissue) and 40.9% (586 µmol CR/g tissue), respectively. Similarly, treatment with *L. paracasei* ATCC 334 reduced lipid and protein oxidation by 36.7% (229 µmol MDA/g tissue) and 45.5% (542 µmol CR/g tissue), respectively. *L. brevis* LBH1070 treatment produced a marked inhibition of lipid peroxidation (51.9%; 174 µmol MDA/g tissue) and a reduction in protein oxidation of 40.3% (592 µmol CR/g tissue). Finally, *L. brevis* LBH1070 synbiotic formulation also significantly decreased lipid and protein oxidation by 46.4% (194 µmol MDA/g tissue) and 34.5% (650 µmol CR/g tissue), respectively, reaching values comparable to those observed in the healthy control group (Figure 6A,B).

These results suggest a partial restoration of the redox balance in popliteal lymph node tissue during experimental arthritis after 21 days of daily treatment, highlighting the antioxidant effects of both probiotic strains and the synbiotic formulation at both the lipid and protein levels.

These findings underscore the antioxidant effect of *L. brevis* LBH1070 on lipid peroxidation in the popliteal lymph node tissue of arthritic mice, surpassing that of the anti-inflammatory drug PBZ. This effect may be attributed to the production of antioxidant metabolites, such as short-chain fatty acids, as well as immunomodulation mediated through the gut microbiota, leading to reduced chronic inflammation [49], which could decrease reactive oxygen species (ROS) generation and, consequently, mitigate oxidative damage to cellular membranes in popliteal lymph node tissue during experimental arthritis, as well as the infiltration of mature immune cells, such as granulocytes, phagocytes, and lymphocytes, into popliteal lymphoid tissue.

Histological analyses corroborated these findings, showing better preservation of tissue structure and fewer lipid vacuoles. Overall, this supports the interpretation of reduced oxidative stress-related damage by PBZ, *L. brevis* LBH1070, *L. paracasei* ATCC 334, and synbiotic treatments. Similarly, previous studies have reported that probiotic strains such as *L. casei*, and *L. acidophilus* significantly decreased lipid peroxidation in a collagen-induced arthritis (CIA) model in Wistar rats [6]. Likewise, in an adjuvant-induced arthritis (AIA) model, lipid and protein peroxidation were reduced in Wistar rats treated with *Bifidobacterium breve* NCIM 5671, *B. longum* NCIM 5672, and *B. bifidum* NCIM 5697 [45]. While these studies did not investigate the antioxidant mechanisms of the strains, the effects were associated with increased activities of catalase, glutathione peroxidase, glutathione reductase, and superoxide dismutase, contributing to reduced oxidative stress during adjuvant-induced chronic inflammation [6,45].

Evaluation of antioxidant enzyme activity revealed that SOD activity in popliteal lymph node tissue from untreated arthritic mice was reduced by 36% (1805 U/g tissue) compared to that observed in the healthy group (2851 U/g tissue), indicating a marked impairment of endogenous antioxidant defense capacity (Figure 6C). PBZ treatment partially restored SOD activity to 60% (2891 U/g tissue) compared to basal levels of the untreated arthritic control group. The other treatments did not show statistically significant differences compared with the arthritic control group (*p* > 0.05); however, a partial recovery was observed for the *L. brevis* LBH1070-treated group (2316 U/g tissue), representing a 28% increase relative to arthritic controls and reaching values comparable to those of the healthy group (*p* > 0.05) (Figure 6C).

SOD is a primary antioxidant defense enzyme that catalyzes the dismutation of superoxide anion (O^2−^) into hydrogen peroxide (H_2_O_2_) and oxygen, constituting the first line of defense against oxidative stress [6]. Higher SOD activity in healthy animals reflects a basal antioxidant state maintained in the absence of severe oxidative stress, which could otherwise impair enzyme structure or expression. In contrast, the reduced SOD activity in arthritic mice may result from enzyme oxidation, depletion, or inactivation caused by sustained oxidative stress during 21 days of chronic inflammation. Because enzyme stability is highly dependent on metal ion availability, excessive cycles of superoxide reaction can progressively degrade SOD, requiring complete resynthesis or regeneration [50]. Similar patterns have been reported in collagen-induced arthritis models in Wistar rats, where probiotic treatment with *L. casei*, and *L. acidophilus* restored SOD activity [6]. In the present study, however, the tested probiotic strains did not enhance SOD activity in vivo, either through gene overexpression or catalytic activity.

Finally, glutathione peroxidase (GPx) activity was significantly higher in the arthritic group (1,258,851 U/g tissue), representing a 1.6-fold increase (65%) compared with healthy mice (763,649 U/g tissue) (Figure 6D). All treatments significantly reduced GPx activity by 29% for PBZ (890,944 U/g tissue), 31.8% for *L. brevis* LBH1070 (859,031 U/g tissue), 35.6% for the synbiotic formulation (810,483 U/g tissue), and 38.2% for *L. paracasei* ATCC 334 (777,330 U/g tissue), compared to the un-treated arthritic control group, and were statistically similar to the healthy control.

GPx catalyzes the reduction of H_2_O_2_, produced by SOD activity, into water using reduced glutathione (GSH) as an electron donor [45]. In arthritis, GPx typically increases initially as a compensatory antioxidant defense but may decrease under prolonged oxidative stress or extensive cellular damage [6]. In this study, GPx activity increased in untreated arthritic controls as a compensatory response, whereas all treatments normalized enzyme activity to basal levels. Previous murine arthritis studies also report GPx reduction relative to healthy controls, followed by significant restoration after probiotic treatment with *Bifidobacterium*, *L. casei*, and *L. acidophilus* [6,45]. Elevated GPx has also been observed in patients with arthritis, both in serum and synovial fluid, often alongside decreases in other antioxidants, suggesting that GPx behavior depends on the stage and severity of the disease [6].

In summary, although the tested probiotic strains did not significantly enhance SOD activity, they effectively reduced oxidative damage and normalized GPx activity, supporting their role in modulating redox balance during experimental arthritis through mechanisms independent of direct antioxidant enzyme induction.

### 3.5. Immunomodulatory Effect

**Figure 7:**
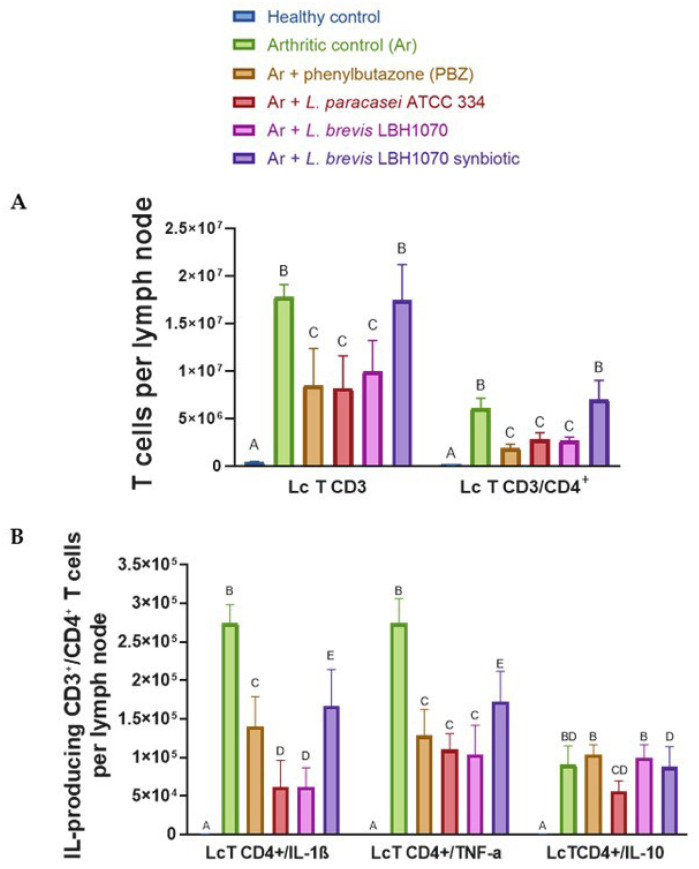
**Effects of treatments on CD3/CD4^+^ T cells and cytokine-producing subsets in popliteal lymph nodes of CFA-induced arthritic mice.** (**A**) T cells, (**B**) IL-producing CD3/CD4^+^ T cells. Data are presented as mean ± standard error of the mean (SEM). Different uppercase letters indicate significant differences among treatments (ANOVA, Student-Newman-Keuls, *p* ≤ 0.05; *n* = 7).

CD3 T cells and the CD3/CD4^+^ T cell subpopulation were significantly increased in untreated arthritic mice (17.80 ± 1.29 × 10^6^ cells/lymph node and 6.06 ± 1.07 × 10^6^ cells/lymph node, respectively) compared with healthy controls (0.38 ± 0.09 × 10^6^ cells/lymph node and 0.11 ± 0.02 × 10^6^ cells/lymph node, respectively) (Figure 7A). Treatment with PBZ significantly reduced total CD3 and CD3/CD4^+^ T cells counted by 52% and 68%, respectively, relative to the arthritic control group (*p* < 0.05). Similarly, probiotic treatments decreased popliteal lymph node infiltration by these immune cell populations. *L. paracasei* ATCC 334 reduced CD3 and CD3/CD4^+^ T cells by 54% and 55%, respectively, whereas *L. brevis* LBH1070 reduced these populations by 44% and 56%. These reductions were statistically comparable to those observed with PBZ treatment. In contrast, the *L. brevis* LBH1070 synbiotic formulation did not significantly affect either CD3 or CD3/CD4^+^ T cell counts compared with the arthritic control group (*p* > 0.05; Figure 7A).

Proinflammatory cytokines produced by infiltrating CD3/CD4^+^ T cells were markedly elevated in arthritic controls compared with healthy mice (Figure 7B). IL-1β-producing CD3/CD4^+^ T cells increased from 0.05 ± 0.01 × 10^4^ cells/lymph node in healthy mice to 29.99 ± 2.43 × 10^4^ in arthritic controls, while TNF-α-producing cells rose from 0.0015 ± 0.0001 × 10^4^ to 27.72 ± 2.53 × 10^4^ cells/lymph node (Figure 7B). PBZ treatment significantly reduced IL-1β- and TNF-α-producing cells by 53% and 54%, respectively. *L. paracasei* ATCC 334 and *L. brevis* LBH1070 reduced, in a statistically similar behavior, Il-1 β and TNF-α-producing cells by 80% and 60%, and IL-1β-producing cells by 79% and 62%, both exceeding PBZ’s inhibitory effect. The synbiotic formulation decreased IL-1β and TNF-α by 44% and 38%, respectively, higher than PBZ but remained lower than free probiotics (Figure 7B).

IL-10-producing CD3/CD4^+^ T cells increased from 0.02 ± 0.01 × 10^4^ in healthy mice to 9.02 ± 2.48 × 10^4^ cells/lymph node in arthritic controls (Figure 7B), and all treatments produced values statistically like those of the untreated arthritic control group (*p* > 0.05).

These changes in lymph node immune profiles support one of the main mechanisms of action of the treatments—immunomodulation during chronic inflammation—which differs from the immunosuppression induced by most conventional anti-arthritic drugs and may be more therapeutically advantageous [51]. PBZ metabolites generated during hepatic biotransformation have shown the capacity to generate blood disorders, such as agranulocytosis and leukopenia, whereas authors propose that these hematotoxic (immunosuppressive) effects are exerted by these metabolites through RNA’s transcription disruption in bone marrow during leukopoiesis, and cell differentiation, following chronic administration of this drug [47]. By modulating immune cell proliferation and maturation, these treatments reduce the severity of chronic inflammation, limiting proinflammatory cytokine and ROS production, and preventing oxidative damage.

These results align with histological and oxidative stress findings and are consistent with previous experimental arthritis studies. For example, 21-day intragastric PBZ administration reduced leukocyte and lymphocyte counts in blood and popliteal lymph nodes of male CD1 mice with CFA-induced arthritis [34]. Oral *Bifidobacterium* for 30 days decreased serum proinflammatory cytokines (IL-1β, IL-6, TNF-α, MCP-1) and increased IL-4 and IL-10 in male Wistar rats with CFA-induced arthritis [45]. Heat-inactivated *Lactobacillus reuteri* administered for 14 days increased CD4+/IL-10+ T cells and reduced IL-6 in female DBA/1J mice with collagen II-induced arthritis [48]. Daily *L. casei* administration for 30 days reduced serum IL-1β, IL-6, IL-17, TNF-α, and IFN-γ in Sprague-Dawley rats with CFA-induced arthritis [44]. *Bacillus coagulans* BACO-17, administered orally for 38 days, reduced TNF-α in cartilage and dose-dependently inhibited IL-1β and IL-6 expression in male BALB/c mice with collagen antibody-induced arthritis [46].

Since arthritis progression involves T lymphocyte (helpers and cytotoxic subpopulations) infiltration into cartilage and subsequent bone destruction mediated by proinflammatory molecules [52], the reductions in CD4+ T cells and proinflammatory cytokines, together with decreased oxidative damage, reinforce the potential immunomodulatory and anti-inflammatory effects of free-form *L. brevis* LBH1070. By modulating chronic immune responses and promoting homeostasis, this strain may serve as a complementary therapeutic strategy for arthritis. However, further preclinical and controlled clinical studies are required to confirm efficacy and establish safe supplementation protocols.

The lower immunoregulatory effect observed with the *L. brevis* LBH1070 synbiotic may relate to murine gastrointestinal physiology. Faster intestinal transit (small intestine: 96 ± 18.4 min; colon: 62.2 ± 21.2 min) [53], shorter absorptive surfaces, higher gastric pH (3–4 vs. 1–2.5 in humans), and simpler intestinal architecture can limit probiotic release from microcapsules, reduce viability, and restrict epithelial interaction [54,55]. These factors may attenuate immunomodulatory effects in mice but are not expected to preclude functionality in humans, where longer transit time, more acidic gastric pH, and more complex intestinal architecture could enable more efficient release and mucosal interaction.

However, it is important to acknowledge a significant limitation of the present study: the absence of direct gut microbiota profiling (e.g., 16S rRNA gene sequencing or shotgun metagenomics). Without empirical fecal or intestinal microbiota data to confirm the colonization dynamics and viability of the administered strains, our hypothesis—that the synbiotic formulation underperformed due to accelerated murine gastrointestinal transit and failed intestinal release—remains speculative. Consequently, future studies incorporating high-throughput sequencing will be essential to accurately characterize the modulatory effects of this synbiotic on the host microbiome and to validate it’s in vivo mechanisms of action [10,49].

Clinical trials are essential, particularly since probiotic mixtures have shown immunoregulatory efficacy in arthritis patients: daily supplementation with *Lactobacillus acidophilus* LA-14, *L. casei* LC-11, *Lactococcus lactis* LL-23, *Bifidobacterium lactis* BL-04, and *Bifidobacterium bifidum* BB-06 for 60 days reduced leukocyte counts, TNF-α, and plasma IL-6 [56]. Similarly, daily *L. casei* 01 for 8 weeks decreased disease activity and serum TNF-α, IL-6, and IL-12 while increasing IL-10 levels and the IL-10/IL-12 ratio [12].

## 4. Conclusions

In this study, *Levilactobacillus brevis* LBH1070, isolated from xastle (pulque) from Milpa Alta, Mexico City, demonstrated an immunomodulatory effect in a CFA-induced experimental arthritis model by significantly reducing CD3^+^ T cells and CD3^+^/CD4^+^ T cells producing IL-1β and TNF-α, surpassing even the effect of phenylbutazone, indicating a potent immunomodulatory action against inflammation. Additionally, it significantly reduced lipid and protein oxidation, thereby reducing oxidative stress and improving membrane integrity in popliteal lymphoid tissue. However, the synbiotic formulation did not overcome the free strain in biological responses, suggesting that, in the murine model used, gastrointestinal physiological conditions such as accelerated transit and reduced absorptive surface may have limited effective probiotic release from the encapsulating matrix. Nevertheless, these limitations do not preclude the functionality of the synbiotic in humans, warranting further investigation. Overall, these results support the potential of *L. brevis* LBH1070 as a viable strain with promising probiotic properties, demonstrating anti-inflammatory, antioxidant, and immunomodulatory effects, and represent an innovative opportunity to utilize native microorganisms in human health. Nevertheless, further studies, including well-controlled clinical trials, are required to validate its efficacy and establish safe and effective protocols for human supplementation.

## Figures and Tables

**Table 1:** **Effect of pH variation on the viability of *Levilactobacillus brevis* LBH107 isolated from xastle (pulque), free and microencapsulated.**

pH Value	pH Tolerance (%)	
	Free *Levilactobacillus brevis* LBH107 Strain	Synbiotic *Levilactobacillus brevis* LBH107 Strain
1	49 ± 0.2 a	53 ± 0.24 a
2	62 ± 0.3 b	68 ± 0.27 b
3	69 ± 0.32 b	70 ± 0.12 b
4	80 ± 0.42 c	82 ± 0.32 c
5	98 ± 0.2 d	98 ± 0.24 d
6	97 ± 0.24 d	99.5 ± 0.32 d
7	96 ± 0.23 d	99.5 ± 0.22 d
8	99 ± 0.11 d	100 ± 0.1 d

Data are shown as the mean (x) with its standard deviation (s.d.) (SDM) from three measurements (*n* = 3). Different lowercase letters indicate significant differences among treatments (Kruskal–Wallis, Dunn, *p* ≤ 0.05).

## Data Availability

The data presented in this study are available on request from the corresponding authors.

## Abbreviations

The following abbreviations are used in this manuscript:

ANOVA	analysis of variance	MD	maltodextrin
APCs	antigen-presenting cells	MDA	malondialdehyde
CFA	complete freund’s adjuvant	NOM	Mexican Official Standard
CFU	colony-forming unit	OD	optical density
CR	reactive carbonyls	PAD	peptidyl arginine deiminase
DMARD	disease-modifying antirheumatic drugs	PAMPs	pathogen-associated molecular patterns
FAO	Food and Agriculture Organization of the United Nations	PBS	phosphate-buffered saline
GPx	glutathione peroxidase	PBZ	phenylbutazone
HLA	human leukocyte antigen	POX	protein oxidation
I	inulin	ROS	reactive oxygen species
i.g.	intragastric	s.c.	subcutaneous
IL	interleukin	SCFAs	short-chain fatty acids
ISAPP	International Scientific Association for Probiotics and Prebiotics	SOD	superoxide dismutase
Lc	lymphocyte	TNF-α	tumor necrosis factor alpha
LPx	lipid peroxidation		
